# Supplementary material for: Memory reactivation during sleep promotes structure abstraction
Source: bioRxiv. 2026 Apr 11:2026.04.10.717748. Preprint. [Version 1] doi: 10.64898/2026.04.10.717748 (PMC13082097; doi:10.64898/2026.04.10.717748)
Supplement: Supplement 1 [file NIHPP2026.04.10.717748v1-supplement-1.pdf]

bioRxiv preprint doi: https://doi.org/10.64898/2026.04.10.717748; this version posted April 11, 2026. The copyright holder for this preprint (which was not certified by peer review) is the author/funder, who has granted bioRxiv a license to display the preprint in perpetuity. It is made available under aCC-BY 4.0 International license.

SUPPLEMENTARY MATERIALS

	Total Sleep	N1	N2	N3	REM
Mean minutes of sleep	90.36 *(23.11)*	14.72 *(9.75)*	51.30 *(20.38)*	13.09 *(10.91)*	11.24 *(11.12)*
Mean proportion of sleep	--	0.18 *(0.14)*	0.57 *(0.17)*	0.14 *(0.11)*	0.11 *(0.11)*
Mean number of cues	66.21 *(37.54)*	0.33 *(2.0)*	29.14 *(24.0)*	35.91 *(35.31)*	0.84 *(5.03)*
Mean proportion of cues	--	0.01 *(0.08)*	0.50 *(0.30)*	0.47 *(0.32)*	0.02 *(0.13)*

Supp. Table 1: Time and cues in different sleep stages. Number and proportion of minutes spent in each sleep stage, and number and proportion of cues played in each sleep stage. Parentheses indicate the standard deviation.

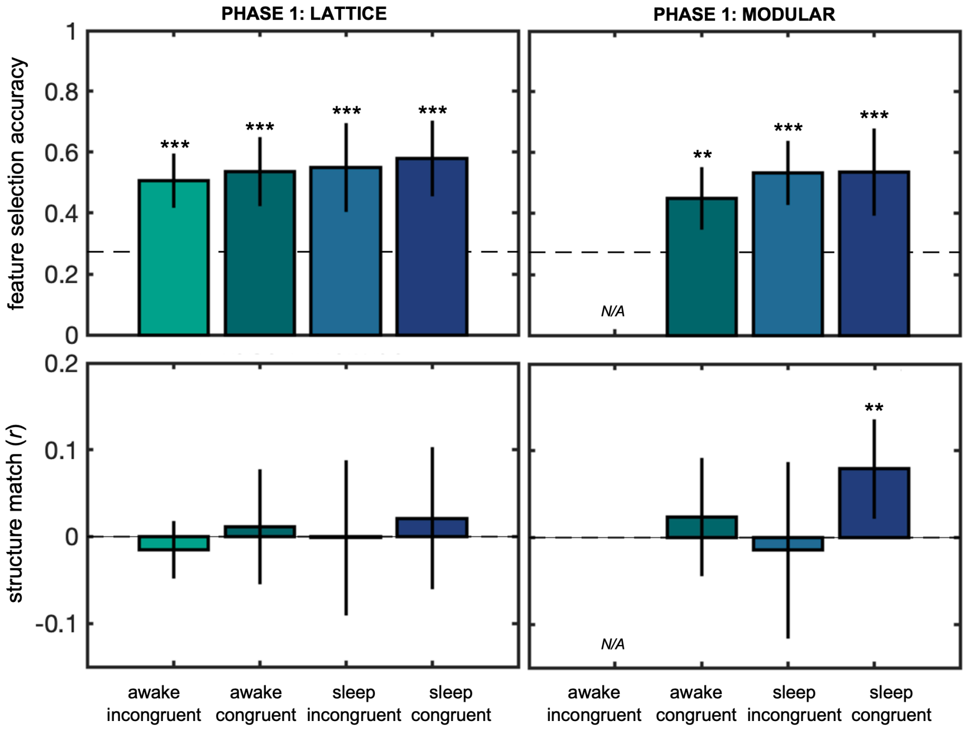

Supp. Figure 1: Memory for Phase 1 categories. Mean feature selection accuracy (top) and structure match data (bottom) for the Phase 1 Lattice (left) and Modular (right) categories. Participants in the awake incongruent condition did not learn a Modular category in Phase 1.

bioRxiv preprint doi: https://doi.org/10.64898/2026.04.10.717748; this version posted April 11, 2026. The copyright holder for this preprint (which was not certified by peer review) is the author/funder, who has granted bioRxiv a license to display the preprint in perpetuity. It is made available under aCC-BY 4.0 International license.

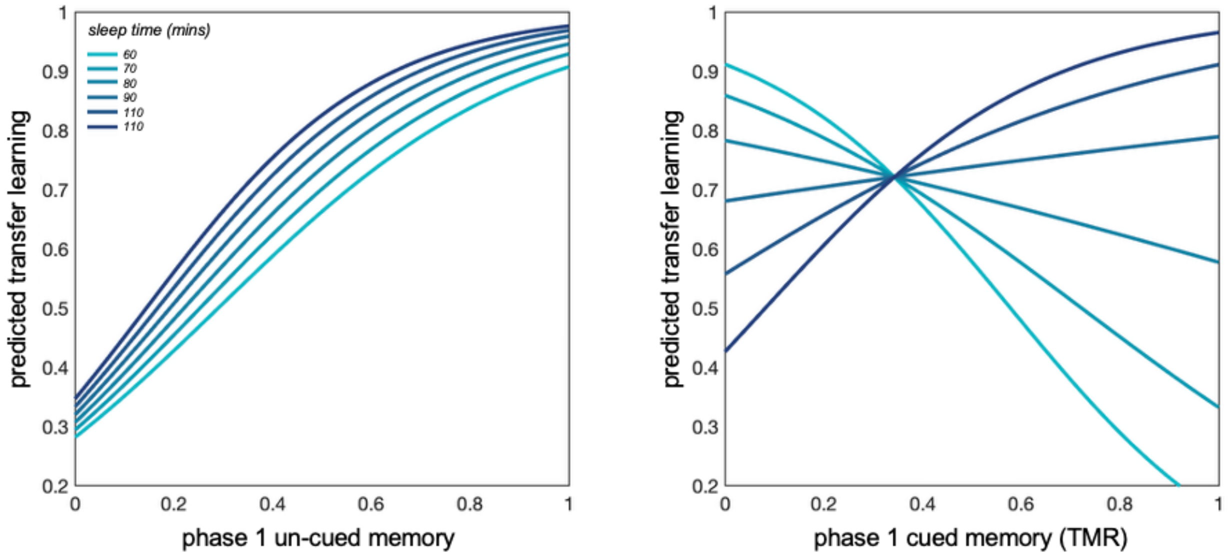

Supp. Figure 2: Sleep interacts with Phase 1 memory to predict transfer learning. Relationships between Phase 1 memory, transfer learning, and sleep were observed in the sleep-congruent group. *Left:* Memory for the un-cued category from Phase 1 was strongly linked to transfer learning performance in Phase 2, and this relationship was not modulated by sleep. *Right:* The correspondence between memory for the cued category in Phase 1 and transfer learning in Phase 2 is modulated by total sleep time. At shorter periods of sleep (~80 mins or less), better cued memory predicts worse transfer learning; as sleep length increases (~90 mins or more), better cued memory predicts better transfer learning performance. These results reveal the potential time course by which surface-level representations are transformed into more abstracted structural representations.

Phase 1 Memory and Sleep Interact to Influence Transfer Learning

In the sleep-congruent group, any benefit of TMR on transfer learning is presumably due to reactivating the memory of the previously learned cued category. We would thus expect a positive relationship between memory for the cued category and transfer learning performance: if the target cues result in transfer, it is because the memory of the Phase 1 category was reactivated, thus this memory should be strengthened. On the other hand, we would expect a weaker relationship between transfer learning and the un-cued category, since the two are not directly causally related. Instead, we found only a marginal relationship between cued memory and transfer learning in the feature selection task ($\chi^2(2, 21)=4.06$, $p=0.053$), but a strong relationship between un-cued memory and transfer ($\chi^2(2, 21)=11.3$, $p=0.001$). Similar results were found when we controlled for total sleep time separately for the cued category ($b=1.84$, $p=0.062$) and the un-cued category ($b=4.16$, $p=0.002$). These puzzling

bioRxiv preprint doi: https://doi.org/10.64898/2026.04.10.717748; this version posted April 11, 2026. The copyright holder for this preprint (which was not certified by peer review) is the author/funder, who has granted bioRxiv a license to display the preprint in perpetuity. It is made available under aCC-BY 4.0 International license.

results suggest a nuanced relationship between memory for the cued category and the transfer learning that this memory reactivation facilitates. To explore this further, we ran an exploratory post hoc analysis testing for a possible interaction with sleep time. The interaction between total sleep time and prior cued memory was significant: when total sleep time, prior cued memory, and their interaction were used to predict transfer learning performance in the feature selection task, there were marginal main effects of prior cued memory (b=-13.27, p=0.054) and total sleep time (b=-0.026, p=0.066), but the interaction between them was significant (b=0.077, p=0.027). The full model including the interaction term explained a significant amount of variance ($\chi^2(2,21)=9.6$, p=0.022) whereas the model excluding the interaction term did not ($\chi^2(2,21)=4.08$, p=0.13). The observed interaction indicates that the correspondence between prior cued memory and transfer learning depends on the amount of sleep (Supp. Fig 2B). When sleep is short (<80 mins), stronger prior cued memory predicts worse transfer learning; however, with longer sleep (>90 mins), the pattern reverses, and stronger prior cued memory predicts better transfer learning. On the other hand, the positive relationship between prior un-cued memory and transfer learning is not modulated by sleep (b=0.012, p>0.7; Supp Fig 2A). Given our previous finding that time spent in N2 or N3 did not impact transfer learning directly, these data potentially reveal the time course by which structural representations are transformed away from surface-level details and into a more abstracted form.

.
